# Plasmid genomic epidemiology of carbapenem-hydrolysing class D β-lactamase (CDHL)-producing Enterobacterales in Canada, 2010−2021

**DOI:** 10.1099/mgen.0.001257

**Published:** 2024-06-19

**Authors:** Nicole Lerminiaux, Robyn Mitchell, Kevin Katz, Ken Fakharuddin, Erin McGill, Laura Mataseje

**Affiliations:** 1 National Microbiology Laboratory, Public Health Agency of Canada, Winnipeg, Manitoba, Canada; 2 Public Health Agency of Canada, Ottawa, Ontario, Canada; 3 North York General Hospital, Toronto, Ontario, Canada

**Keywords:** β-lactamase, antimicrobial resistance, carbapenemase, epidemiology, genomics, plasmid, surveillance

## Abstract

Carbapenems are last-resort antibiotics for treatment of infections caused by multidrug-resistant *Enterobacterales*, but carbapenem resistance is a rising global threat due to the acquisition of carbapenemase genes. Oxacillinase-48 (*bla*_OXA-48_)-type carbapenemases are increasing in abundance in Canada and elsewhere; these genes are frequently found on mobile genetic elements and are associated with specific transposons. This means that alongside clonal dissemination, *bla*_OXA-48-type_ genes can spread through plasmid-mediated horizontal gene transfer. We applied whole genome sequencing to characterize 249 *bla*_OXA-48-type_-producing *Enterobacterales* isolates collected by the Canadian Nosocomial Infection Surveillance Program from 2010 to 2021. Using a combination of short- and long-read sequencing, we obtained 70 complete and circular *bla*_OXA-48-type_-encoding plasmids. Using MOB-suite, four major plasmids clustered were identified, and we further estimated a plasmid cluster for 91.9 % (147/160) of incomplete *bla*_OXA-48-type_-encoding contigs. We identified different patterns of carbapenemase mobilization across Canada, including horizontal transmission of *bla*_OXA-181_/IncX3 plasmids (75/249, 30.1 %) and *bla*_OXA-48_/IncL/M plasmids (47/249, 18.9 %), and both horizontal transmission and clonal transmission of *bla*_OXA-232_ for *Klebsiella pneumoniae* ST231 on ColE2-type/ColKP3 plasmids (25/249, 10.0 %). Our findings highlight the diversity of OXA-48-type plasmids and indicate that multiple plasmid clusters and clonal transmission have contributed to *bla*_OXA-48-type_ spread and persistence in Canada.

## Data Summary

Raw sequencing reads were deposited to the NCBI SRA archive under BioProject PRJNA855907 (https://www.ncbi.nlm.nih.gov/bioproject/?term=PRJNA855907); see Table S1, available in the online version of this article for a list of accessions. Complete *bla*_OXA-48-type_-encoding plasmid sequences were deposited to NCBI GenBank under the accessions listed in Table S2.

**Impact Statement**

Resistance to last-resort carbapenem antibiotics is a global public health threat. The dissemination of carbapenemase resistance genes is significantly influenced by plasmids, which are mobile genetic elements that can transfer between unrelated species and strains. Consequently, understanding the features and distribution of carbapenemase-encoding plasmids is crucial for pathogen surveillance and mitigation of resistance. In this work, we used long-read and short-read sequencing to characterize genomic epidemiology of OXA-48-type carbapenemase-encoding plasmids across more than a decade of *Enterobacterales* surveillance data in Canada. Examining the genetic and genomic context of OXA-48-type carbapenemases reveals trends in the plasmid reservoir in Canada and enhances future international pathogen surveillance.

## Introduction

Carbapenems are one of the last resort antibiotics for treating serious infections caused by multidrug-resistant Gram-negative bacteria. Carbapenem-resistant pathogens have emerged following the clinical use of carbapenems, and they pose a significant threat to human health [1]. Carbapenem-resistant *Enterobacterales* have been reported worldwide largely as a consequence of acquisition of carbapenemase genes [2,3].

Of the different classes of carbapenemases, the oxacillinase-48 (OXA-48)-type carbapenemases are one of the most commonly identified in many countries, including Canada [3,7]. OXA-48-type carbapenemases encompass several different *bla*_OXA-48-type_ variants, including *bla*_OXA-48_, *bla*_OXA-181_, *bla*_OXA-232_, and *bla*_OXA-204_. These carbapenemases are clinically significant as they have activity against penicillins, narrow-spectrum cephalosporins, carbapenems, and provide resistance to many β-lactam inhibitors [8,9]. The first description of OXA-48-type producers in Canada was in 2011 wherein four patients harboured distinct species and sequence types with either *bla*_OXA-48_ or *bla*_OXA-181_ [10]. In recent years, the proportion of OXA-48-type-producing organisms in Canada has increased from 12.4 % (*n*=20) of carbapenem-resistant organisms isolated in 2016 to 21.4 % in 2020 (*n*=45) [7].

Clonal transmission of high-risk organisms carrying *bla*_OXA-48-type_ genes on persistent plasmids has played an important role in the dissemination of OXA-48-type carbapenemases. High-risk *Klebsiella pneumoniae* ST147, ST307, ST15, ST14 and *E. coli* ST410, ST38 clonal lineages have been linked to clonal OXA-48-type outbreaks [8,11, 12]. For example, *Esherichia coli* ST38 encoding a chromosomal *bla*_OXA-48_ carbapenemase was found in 25 different hospitals sites around the United Kingdom [12]. The dominant sequence types and species carrying OXA-48-type carbapenemases in Canada between 2011 and 2014 were ST38 and ST410 in *Escherichia coli* and ST14 in the *K. pneumoniae* species complex [13]. OXA-48-type variants have also been described in *Citrobacter, Enterobacter, Serratia,* and *Providencia* species [5,8].

The global ascendency of OXA-48-type carbapenemases can be largely explained by horizontal plasmid spread [8]. As with many types of antimicrobial resistance genes, *bla*_OXA-48-type_ genes are frequently found on mobile genetic elements that can transfer between strains, species, and genera [4,14]. In Canada and elsewhere, *bla*_OXA-48_ is associated with IncL/M plasmids, *bla*_OXA-181_ is associated with IncX3 plasmids, and *bla*_OXA-232_ is associated with ColKP3/ColE2-type plasmids [8,13, 15,17]. Certain plasmid types have also been associated with specific species and sequence types [8]; for example, *bla*_OXA-232_ on ColKP3 plasmids has been associated with *K. pneumoniae* ST231 in India [18]. Each of the common OXA-48-type-encoding plasmids are additionally associated with different types of transposons which can further contribute to their mobilization [8,14]. For example, Tn*1999* is associated with *bla*_OXA-48_ on IncL/M plasmids and several structural variants have been described [8,19]. Similarly, Tn*2013* is associated with both *bla*_OXA-181_ on IncX3 plasmids and *bla*_OXA-232_ on ColKP3/ColE2-type plasmids, although the transposon structure is truncated on IncX3 plasmids [8,20].

Here, we applied whole genome sequencing to characterize the molecular epidemiology of OXA-48-type carbapenemase-producing isolates collected by the Canadian Nosocomial Infection Surveillance Program from 2010 to 2021. Using combined short- and long-read sequencing of selected representatives to generate complete *bla*_OXA-48-type_-encoding plasmids, we investigated the diversity of carbapenemase-encoding plasmids among these isolates across Canada and compared them to the global context of *bla*_OXA-48-type_ genes.

## Methods

### Surveillance period and PCR confirmation of bla_OXA-48-type_carbapenemase gene

The Canadian Nosocomial Infection Surveillance Program (CNISP) is a sentinel surveillance system which collects epidemiological and linked microbiology data from 90 Canadian acute-care hospitals across ten provinces and one territory. *Enterobacterales* organisms isolated from patients between 2010 and 2021 were eligible for inclusion by minimum inhibitory concentration (MIC) above clinical breakpoints [21] or if they tested positive using molecular (PCR) or phenotypic testing (mCIM, CARBA-NP) [22]. Serial isolates from the same patient were included if the organism or carbapenemase gene differed, and there were no restrictions on collection from particular body sites. Further information about CNISP can be found online (https://health-infobase.canada.ca/cnisp/). Eligible isolates were submitted to the National Microbiology Laboratory (NML; Winnipeg, Canada) by Canadian hospitals and provincial public health laboratories for *bla*_OXA-48-type_ carbapenemase gene confirmation which was conducted by multiplex PCR as previously described [13]. A total of 249 isolates encoding *bla*_OXA-48-type_ carbapenemases were collected from 2010 to 2021 from 33 hospital sites (Table S1), with one hospital submitting 19 % (47/249) of all isolates. Where applicable, the Central region refers to the provinces of Ontario and Québec, and the West region refers to the provinces of British Columbia, Alberta, Saskatchewan, and Manitoba. Only a few cases were detected in the East region (provinces of Nova Scotia, New Brunswick, Prince Edward Island, and Newfoundland and Labrador) so these were grouped in Central region for epidemiological investigation.

### Species complex definitions

Organism genus was confirmed using the RefSeq Masher Matches tool [23]. We used the following definitions for species complexes: the *K. pneumoniae* species complex includes *K. pneumoniae*, *K. quasipneumoniae*, and *K. variicola* [24]; the *Enterobacter* cloacae complex includes *E. cloacae, E. hormachei, E. asburiae, E. kobei*, and *E. ludwigii* [25]; and the *Citrobacter freundii* complex includes *C. freundii, C. portucalensis, C. werkmanii*, and *C. youngae* [26]. To assign species, Kleborate v2.2.0 [24] was used for species identification of *Klebsiella* with default parameters. Genomic clades and clusters in the *Enterobacter* spp. were defined by pairwise average nucleotide identity-based distance matrix using FastANI v1.3 [27,28] and the clade was assigned when the mean average nucleotide identity value was >95 %.

### Whole genome sequencing and assembly

All 249 isolates were sequenced with Illumina MiSeq platforms and 88 of these were additionally sequenced using Oxford Nanopore Technologies (ONT). Isolates for ONT long-read sequencing represented about 33 % (88/249) of all OXA-48-type cases and isolates were selected to maintain representative proportions of each province in Canada. Genomic DNA was extracted using Epicentre MasterPure Complete kits (Mandel Scientific, Guelph, ON, Canada). The same DNA extract was used for both short-read Illumina and long-read ONT sequencing where possible. Short-read libraries were created with TruSeq Nano DNA HT sample preparation kits (Illumina, San Diego, CA, USA). Paired-end, 301 bp indexed reads were generated on an Illumina MiSeqTM platform (Illumina). Long-read sequences were generated using the Rapid Barcoding Kit (SQK-RBK004) or the Rapid Barcoding Kit 96 (SQK-RBK110.96) on R9.4.1 flow cells with the MinION Mk1B (ONT, Oxford, Oxfordshire, UK). Read data was basecalled and demultiplexed with Guppy v6.3.7 using the Super High Accuracy model (ONT). Average Illumina depth of coverage was 105X and average ONT depth of coverage was 72X.

### Bioinformatic analyses

The assembly workflow was managed using Snakemake [29]. ONT reads were trimmed with Porechop v0.2.3_seqan2.1.1 [30] and filtered for Q-score>10 and length>1000 bases with Filtlong v0.2.1 [31]. Illumina reads had adaptors trimmed and were filtered for an average Q-score>30 with trim-galore v0.6.7 [32]. FastQC v0.11.9 [33] and Nanoplot v1.28.2 [34] were used to assess quality control metrics for Illumina and ONT reads respectively. Isolates with Illumina-only data were assembled with Unicycler v0.5.0 using default settings [35]. Those with Illumina and ONT data were assembled with Flye v2.9.2 [36], Raven v1.8.1 [37], and Miniasm v0.3_r179 [38], with the consensus assembly generated by Trycycler v0.5.0 [39]. A subset of these failed to generate a consensus with Trycycler (low ONT coverage, incomplete chromosomes) so were assembled using hybrid Unicycler v0.5.0 with default settings. Assemblies were polished with short reads using Polypolish v0.5.0 [40] and POLCA from MaSuRCA v4.0.9 [41]. A total of 70 OXA-48-type plasmids were circularized from these assemblies (Table S2).

StarAMR v0.9.1 [42] was used to detect antimicrobial resistance genes using the ResFinder database v2022-05-24 [43] and sequence type using the MLST database v2.23.0 [44,45]. Panaroo v1.3.2 [46] was used to estimate the pangenome. Plasmid taxonomic unit (PTU) designations were obtained from COPLA [47]. The MOB-typer tool from MOB-suite v3.1.4 [48,49] was used to identify plasmid replicons and mobility class using the default databases. The dnadiff tool which is part of MUMmer v3.23 [50] was used to align plasmid sequences to reference transposons (Table 1) with default settings and ISFinder database version 25 July 2023 [51] was used to delineate boundaries of insertion elements. SNVPhyl v1.2.3 [52] was used to investigate single nucleotide variants within primary plasmid clusters using the following parameters: min_coverage=10, min_mean_mapping=30, SNV_abundance_ratio=0.75, density threshold cutoff=2, size of search window=20. PHASTER (prophage/virus database version: 22 December 2020) was used to check for known prophages [53].

**Table 1.** Features of the complete OXA-48-type Canadian plasmids and their transposons

Plasmid cluster	*bla*_OXA-48-type_ gene (n/N)*	Tn type	Tn subtype (n/N)*	Plasmid and Tn reference
AA836 (IncX3/rep_cluster_1195)	181 (19/20)	Tn*2013*	Tn*2013* (19/20)	KP400525.1
	1181 (1/20)	Tn*2013*	Tn*2013* (1/20)	KP400525.1
AB871 (IncL/M)	48 (14/14)	Tn*1999*	invTn*1999.1* (1/14)	AY236073.2
			Tn*1999.2* (2/14)	JN714122.1
			invTn*1999.2* (10/14)	JN714122.1
			Other (1/14)	
AB484 (rep_cluster_1195)	181 (2/17)	Tn*2013*	truncTn*2013* (1/17)	JX423831.1
			IS*Kpn26* insertion (1/17)	
	232 (15/17)	Tn*2013*	truncTn*2013* (14/17)	JX423831.1
			IS*Kpn26* insertion (1/17)	
AC843 (IncC/IncF)	204 (4/4)	Tn*2016*	Tn*2016* intact IS*Ecp1* (4/4)	CP047276.1
Chromosome	48 (9/27)	Tn*6237*	Tn*6237* (2/9)	KT444705.1
			truncTn*6237* (6/9)	
			Other (1/9)	
	204 (4/27)	prophage	*C. freundii* prophage	MG430338.1
	244 (12/27)	Tn*51098*	Tn*51098* (2/12)	KR364794.1
			truncTn*51098* (10/12)	
	181 (2/27)	Tn*2013*	truncTn*2013* (2/2)	JX423831.1

1*n/N indicates number of complete and circular Canadian plasmids with this feature (n) out of the total number of Canadian plasmids in the respective cluster (N).

Plots were created using R v4.3.0 [54] and the following packages: tidyverse packages [55], patchwork v1.1.2 [56], and ggpubr v0.6.0 [57].

### Plasmid clustering and containment analysis

MOB-suite primary cluster designations are a useful way to broadly cluster plasmids for epidemiologically studies, and so plasmids assigned to different primary MOB-clusters are sufficiently unrelated to not be considered as part of an epidemiologically relevant transmission event [48,49]. However, plasmids that share the same primary cluster designation can be examined in more detail through higher resolution subtyping such as secondary cluster designations. If two plasmids are assigned to the same secondary cluster, they have near duplicate sequences and are sufficiently related to be strong candidates for outbreak investigations [48,49]. In addition to secondary cluster designation, epidemiological data is required to best assess direct plasmid transmission.

For plasmid clustering analysis, the PLSDB v2021_06_23v2 [58] database was downloaded and clustered alongside the 70 circular OXA-48-type plasmids in this study using MOB-cluster from the MOB-suite v3.1.4 package [48,49]. The primary and secondary cluster numbers generated here are unique to this manuscript and differ from those used in the default MOB-suite database. For plasmid containment analysis, all 575 circular plasmids completed in this study (including the 70 OXA-48-type plasmids) were clustered using MOB-cluster to create a custom Canadian plasmid database. All 249 isolates were screened for plasmids with MOB-recon using this custom database and the output was filtered to focus on the reconstructed plasmids containing *bla*_OXA-48-type_ genes.

## Results

### Characteristics of Canadian OXA-48-type carbapenemase-producing isolates

**Fig. 1.**
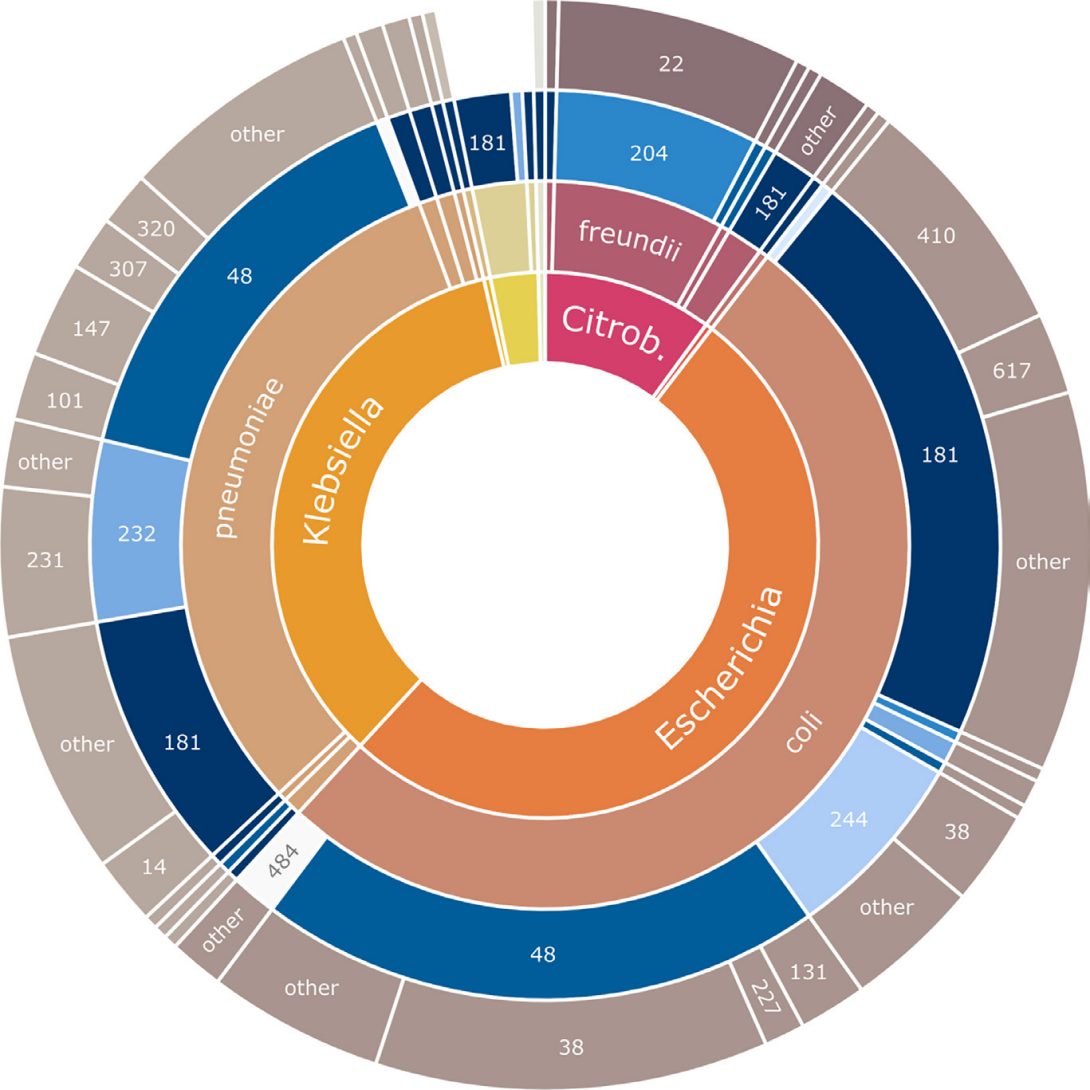
Genera (inner ring), species (inner-middle ring), *bla*_OXA-48-type_ gene (outer-middle ring), and multi-locus sequence type (MLST; outer ring) of *bla*_OXA-48-type_-encoding isolates in this study (249 total isolates). MLST profiles found in two or fewer isolates were grouped in to ‘other’. Not all labels are displayed. ‘Citrob.’ is abbreviation for *Citrobacter*.

A total of 249 nosocomial OXA-48-type-producing isolates were submitted by 33 hospital sites across Canada (21 in the Central region, 12 in the West region) from 2010 to 2021 (see Methods for more detail) (Table S1). We performed short-read sequencing on all isolates and long-read sequencing on a selection of 88 isolates. The OXA-48-type-producing isolates belong to seven genera and twelve species, with the most common genera being the *E. coli* (128/249, 51.4 %), *K. pneumoniae* species complex (83/249, 33.3 %), and *C. freundii* complex (23/249, 9.2 %) (Fig. 1). The most common sequence types within species were *E. coli* ST38 (38/128, 30.0 %), *E. coli* ST410 (23/128, 18.0 %), *C. freundii* ST22 (18/23, 78.2 %), *K. pneumoniae* ST231 (11/83, 13.2 %), and *K. pneumoniae* ST147 (9/83, 10.8 %).

**Fig. 2.**
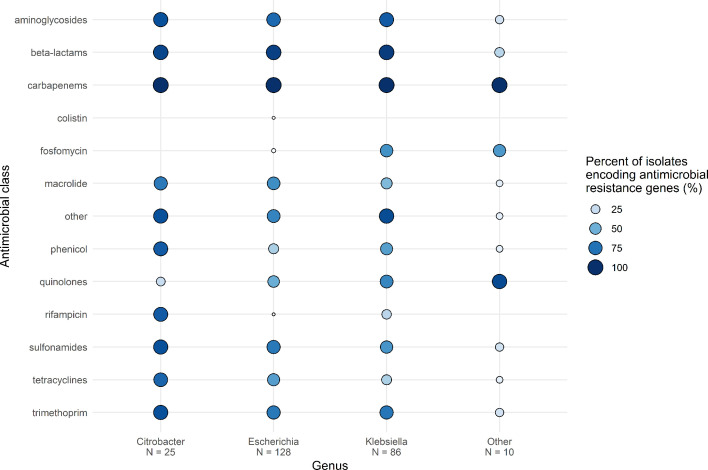
Proportion of isolates encoding antimicrobial resistance genes identified by StarAMR then categorized by drug class presented across the top three genera. Values represent the proportion of isolates encoding genes belonging to a certain antimicrobial class. ‘N =’ indicates the number of isolates in that genus. ‘Other’ genus includes *Enterobacter* spp.*, Providencia* spp.*, Raoultella* spp.*,* and *Shigella* spp.

Using the StarAMR tool for the detection of resistance genes in the whole genome sequencing data, we observed 92.0 % (229/249) of isolates harboured additional β-lactamase genes alongside the *bla*_OXA-48-type_ genes (Fig. 2). Of the 229 isolates harbouring additional β-lactamases, *bla*_TEM-1B_ (116/229, 50.7 %), *bla*_CTX-M-15_ (112/229, 48.9 %), *bla*_OXA-1_ (86/229, 37.6 %), *bla*_SHV-100_ (25/229, 10.9 %), and *bla*_CMY-4_ (21/229, 9.2 %) were the most common types. Sulfonamide, macrolide, quinolone, and aminoglycoside resistance genes were commonly observed among multiple genera; the most common genes included *sul1* (137/249, 55.0 %), *mph(A*) (135/249, 54.2 %), *qacE* (135/249, 54.2 %), *qnrS1* (108/249, 43.4 %), *aph*(6)-Id (103/249, 41.4 %), *aph*(3’)-Ib (102/249, 41.0 %), *sul2* (101/249, 40.6 %)*,* and *aac*(6’)-Ib-cr (99/249, 39.8 %). *Klebsiella* spp. and *E. coli* were equally likely to encode aminoglycoside or additional β-lactamase genes (*P*>0.05, 84 % vs 78% and 95 % vs 94 %). Fosfomycin resistance genes were more likely to be found in *Klebsiella* spp. than *E. coli* (*P*<0.001, 63 % vs 2 %), whereas tetracycline resistance genes were significantly more likely to be found in *E. coli* than *Klebsiella* spp. (*P*<0.001, 58 % vs 34 %).

A total of 259 *bla*_OXA-48-type_ genes were detected among the 249 isolates, indicating an occurrence of 3.6 % of isolates habouring two copies of *bla*_OXA-48-type_ genes. The *bla*_OXA-181_ variant was the most common (101/259, 39.0 %), followed by *bla*_OXA-48_ (92/259, 35.5 %), *bla*_OXA-204_ (22/259, 8.5 %), *bla*_OXA-232_ (20/259, 7.7 %), *bla*_OXA-244_ (19/259, 7.3 %), *bla*_OXA-484_ (5/259, 1.9 %), and *bla*_OXA-1181_ (1/259, 0.4 %).

Certain *bla*_OXA-48-type_ genes were associated with certain sequence types (ST) (Fig. 1). *bla*_OXA-181_ was found in diverse *E. coli* and *K. pneumoniae* species complex ST backgrounds, however we noted that *bla*_OXA-181_ was associated with *E. coli* ST410 (17/92, 18.5 %). In contrast, *bla*_OXA-232_ was found mainly in *Klebsiella* species with a large proportion from ST231 (11/19, 57.9 %). *bla*_OXA-204_ was found exclusively in *C. freundii* ST22 all isolated from a single hospital site. We also noted that *E. coli* ST38 comprised 47.4 % (9/19) and 31.5 % (29/92) of reported *bla*_OXA-244_ and *bla*_OXA-48_ genes, respectively.

### Situating the complete and circular bla_OXA-48-type_ Canadian plasmids within a global plasmid dataset

**Fig. 3.**
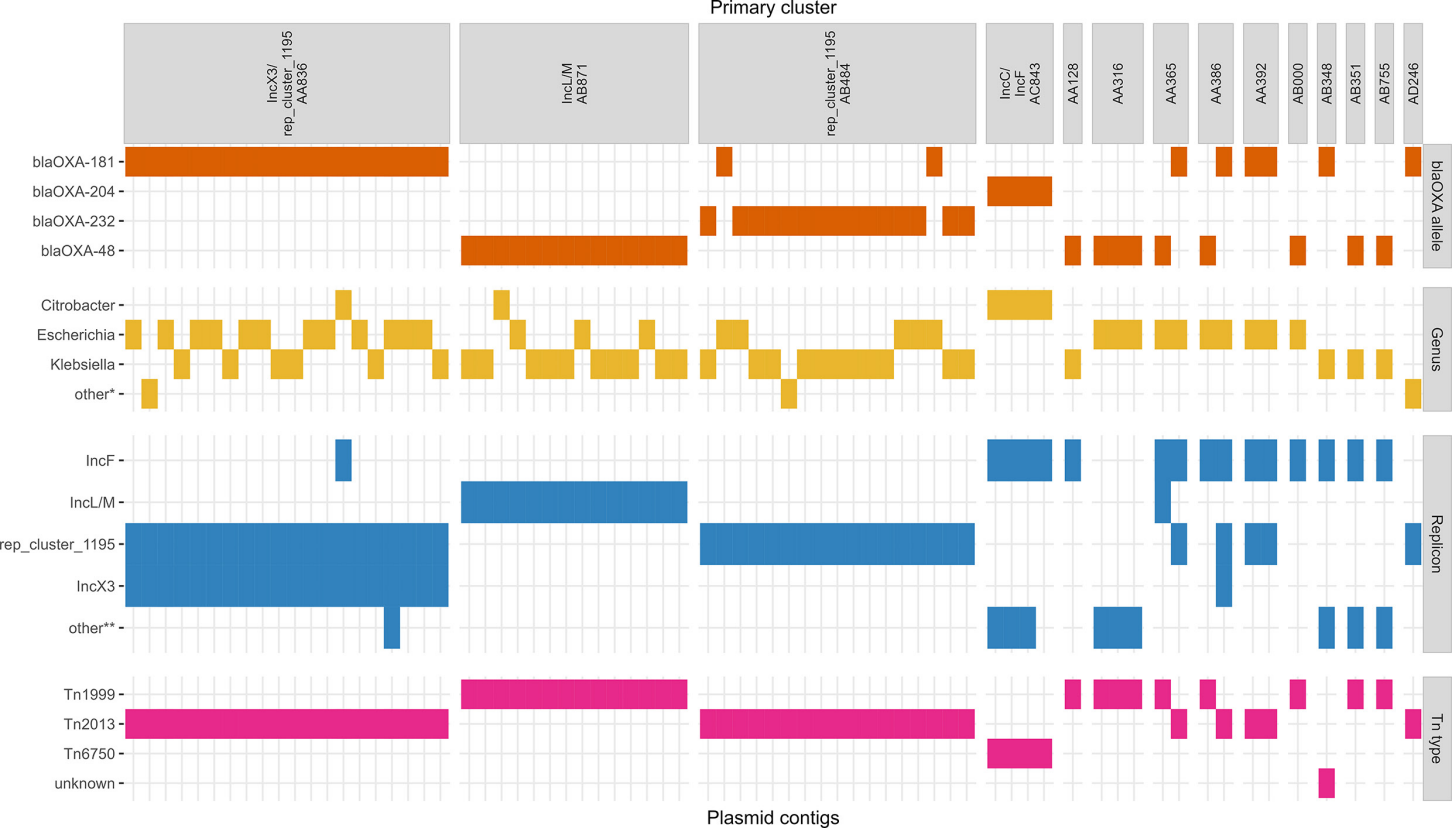
Characteristics of 70 complete OXA-48-type plasmids sequenced in this study. Groups on the x-axis correspond to primary cluster IDs generated by *de novo* clustering of the global dataset (PLSDB and Canadian OXA-48-type plasmids). Tn type indicates transposon type. other* indicates *Enterobacter, Providencia, Raoultella,* and *Shigella* genera; other** indicates rep_cluster_1418, IncHI1B, and ColKP3 replicons; unknown indicates an uncharacterized or truncated transposon.

Of 249 sequenced isolates we obtained only two closed circular plasmids from Illumina-only assemblies. Using a hybrid assembly approach on a subset of 88 isolates, we obtained an additional 68 OXA-48-type complete circular plasmids and identified *bla*_OXA-48-type_ genes on 27 complete chromosomes. Complete OXA-48-type plasmids (*n*=70) ranged from 6.1 kb to 364.4 kb and were distributed among the top species as described above (Fig. 3 and Table S2). Common plasmid incompatibility groups included rep_cluster_1195 (also known as ColKP3 in PlasmidFinder [59] or ColE2-type [8]) (42/70, 60.0 %), IncX3 (21/70, 30.0 %), IncL/M (15/70, 21.4 %), IncF-type (16/70, 22.9 %), with half of the plasmids containing two or more replicons (35/70, 50.0 %). Plasmids were predicted to be conjugative (48/70, 68.6 %), mobilizable (20/70, 28.5 %), or non-mobilizable (2/70, 2.9 %) based on the presence of mating pair formation (MPF) proteins, relaxases, and self-encoded *oriT* sequences detected by MOB-suite [48].

To investigate where these plasmids fit within the global context, we clustered our 70 complete plasmids alongside 34 513 plasmids present in PLSDB [58] using the MOB-cluster tool from MOB-suite [48,49], referred to hereafter as the global dataset. Our OXA-48-type plasmids grouped into a subset of 14 primary clusters, all of which contained representative plasmids from the global dataset. Primary clusters are defined as having a pairwise Mash distance of <0.06, and can contain multiple secondary clusters with a pairwise Mash distance of <0.025.

**Fig. 4.**
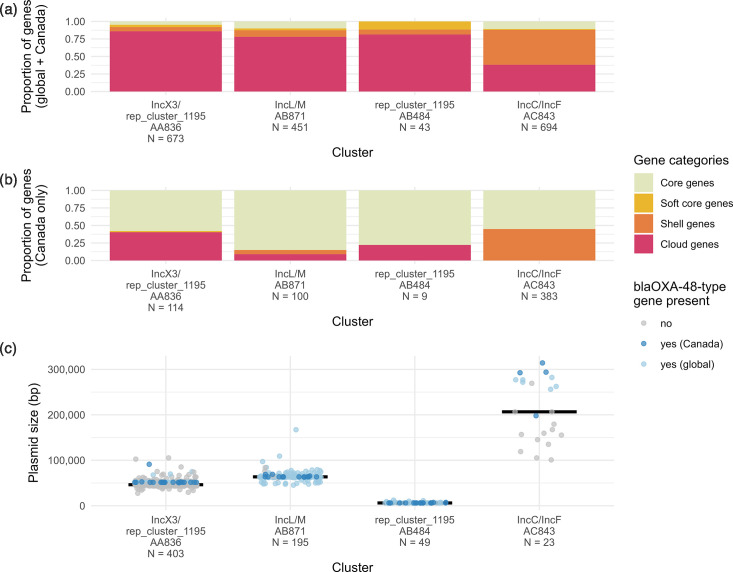
Pangenome size and *bla*_OXA-48-type_ prevalence among top four MOB-suite primary clusters identified among the global plasmid dataset (PLSDB and Canadian OXA-48-type plasmids). Pangenome size was calculated for (**a**) the global plasmid dataset (Canadian and PLSDB) and (**b**) Canadian plasmids only. Gene categories represent genes found in 99–100 % of plasmids (core), 95–99 % of plasmids (soft core), 15–95 % of plasmids (shell) and 0–15 % of plasmids (cloud), and N indicates the total number of genes identified per cluster. (**c**) Plasmid length in base pairs and prevalence of *bla*_OXA-48-type_ genes among plasmids in each primary cluster in the global dataset. N indicates the total number of plasmids per cluster.

We observed that four primary clusters represented 78.6 % (55/70) of Canadian plasmids and tightly clustered (Mash distance <0.025) with other known OXA-48-type plasmids isolated from multiple genera in the global dataset (Fig. 4 and Table 2). Within the global dataset, the presence of *bla*_OXA-48-type_ alleles varied between the top primary clusters. Of the top primary clusters, *bla*_OXA-48-type_ genes were found on 96.9 % (primary cluster AB871), 95.9 % (primary cluster AB484) and 22.3 % (primary cluster AA836) of plasmids, respectively. In three of four top primary clusters, plasmids carried only a few antimicrobial resistance genes (averages of 1.1, 1.5, and 1.7 resistance genes per plasmid); primary cluster AC843 was an exception with an average of 12.4 resistance genes per plasmid, although most of these plasmids (21/23, 91.3 %) were harboured by *Citrobacter* species indicating this may be a clonal trend. The percentage of core/soft core genes (defined as genes found in >95  % of plasmids) varied from 8 % (AA836) to 13 % (AB871). Primary cluster AA836 had the broadest host range with plasmids being found in twelve genera, whereas primary cluster AC843 plasmids were only found in three genera to date.

**Table 2.** Summary features of top four primary plasmid clusters containing Canadian OXA-48-type plasmids and PLSDB plasmids from the global dataset

Primary cluster ID	Num. Canadian plasmids	Median size (kb)	Predicted mobility*,†	Replicon type*,†	Relaxase type*,†	MPF type*,†	Number of genera	Core genes (%)§	*bla*_OXA-48-type_ allele*	*bla*_OXA-48-type_ freq	Mean number of ARGs per plasmid‡	Plasmid Taxonomic Unit (PTU)*,¶
AA836	20	46.3	conjugative	IncX3/rep_cluster_1195	MOBP	MPF_T	12	0.08	*bla*_OXA-181_	0.22	1.5	PTU-X3
AB871	14	63.6	conjugative	IncL/M	MOBP	MPF_I	6	0.13	*bla*_OXA-48_	0.97	1.7	PTU-L/M
AB484	17	6.1	mobilizable	rep_cluster_1195	MOBP	None	4	0.12	*bla*_OXA-232_	0.96	1.1	PTU-E37
AC843	4	206.6	conjugative	IncC,IncFIB,IncFIC,IncFII	MOBF	MPF_F	3	0.12	*bla*_OXA-204_	0.43	12.4	PTU-C

1v*Values indicate the most common genotype in the cluster and may not apply to all plasmids in the cluster.

2 †vValues obtained from MOB-suite. Mobility is assigned based on presence of relaxase (mobilizable) and/or MPF proteins (conjugative) or absence of both (non-mobilizable).

3‡ARGs=antimicrobial resistance genes, including *bla*_OXA-48-type_ genes.

4§Core genes represents the number genes present in >95 % of plasmids in the cluster, divided by the total number of non-redundant genes in the cluster.

5¶PTU values obtained from COPLA.

All top primary plasmid clusters except for AB484 contained plasmids encoding one or multiple genes involved in stability/transfer/defence to support their persistence in the host cell. Primary cluster AB871 plasmids encoded two plasmid partition stability genes (*parM* and an unnamed plasmid family stability protein), as well as the *pemKI* toxin-antitoxin system. All plasmids in this primary cluster also encode the *relB* antitoxin. Most plasmids encoded *ssb*, the single-stranded binding protein (175/195, 89.7 %), and most also encoded an antirestriction gene (174/195, 89.2 %). All but one plasmid in primary cluster AC843 (22/23, 95.7 %) encoded two plasmid partition stability proteins (both labelled *parB*), plasmid SOS inhibition genes *psiA* and *psiB*, an antirestriction gene, a single-stranded binding protein *ssb,* and the EcoRII restriction modification system (including both the restriction enzyme and methyltransferase). Primary cluster AA836 encoded a *parA* partition gene in 99.3 % (*n*=400/403) of plasmids. No other stability/transfer/defence genes were detected in >10 % of plasmids in the top primary clusters.

The remaining nine primary clusters contained two or fewer Canadian plasmids per cluster, which suggests that they are not dominant in the Canadian population of OXA-48-type plasmids. These typically had IncF-type replicons that often appeared to be hybrids with IncH, IncL/M, IncX3, rep_cluster_1195, or other replicons, and the majority (10/15, 66.7 %) were predicted to be conjugative due to the presence of relaxases and mating pair formation proteins. Given the high proportion of conserved genes among Canadian plasmids within each top primary cluster (Fig. 4), we focused on the features of the Canadian plasmids separately from plasmids in global dataset in more detail in the next section.

### Canadian plasmids in primary cluster AA836: bla_OXA-181_ on IncX3/rep_cluster_1195 replicons

The Canadian plasmids in primary cluster AA836 encoded two replicons (IncX3 and rep_cluster_1195) and were classified into two secondary clusters: AM772 (19/20) and AM770 (1/20). Plasmids in secondary cluster AM772 were 51.5 kb in length and were isolated from *K. pneumoniae* species complex, *K. oxytoca, E. coli*, *Citrobacter werkmannii*, and *Raoultella ornithinolytica* between 2015 and 2020 from sixteen hospital sites in four provinces. These plasmids were >99.99 % identical and had 0–4 SNVs relative to the first reported case of *bla*_OXA-181_ on an IncX3/rep_cluster_1195 plasmid from China (pOXA181_EC14828, KP400525.1) [20]. One of these plasmids contained a SNV in *bla*_OXA-181_ which corresponded to *bla*_OXA-1181_. In addition to *bla*_OXA-181_ in Tn*2013*, the only other antimicrobial resistance gene found on these plasmids was *qnrS1*. The plasmid assigned to secondary cluster AM770 is a hybrid plasmid which contains the 51.5 kb IncX3/rep_cluster_1195 replicon along with an additional IncFIB replicon and MOBC relaxase for a total size of 91.2 kb. This plasmid encodes two aminoglycoside resistance genes along with *bla*_OXA-181_ and *qnrS1*. The prevalence of secondary cluster AM772 plasmids among multiple genera and provinces along with the presence of conjugation genes (MOBP relaxase and MPF_T mating pair formation protein) suggest that horizontal transmission has contributed to these plasmids’ persistence in Canada. *bla*_OXA-181_ is known to be associated with the truncated Tn*2013* transposon in IncX3/rep_cluster_1195 plasmids alongside *qnrS* and several IS elements: IS*3000*, IS*Kpn19*, IS*26*, and IS*2*-type [8,20]. All twenty Canadian plasmids in primary cluster AA836 have this same Tn*2013* structure [20] (Table 1).

We examined the location of the SNVs in the secondary cluster AM772 plasmids in our dataset relative to the reference plasmid KP400525.1 [20] and did not observe any notable trends. The SNVs (11 in total) are located in several transposases (IS*Kox3*, IS*3000*, and IS*Kpn19*), *bla*_OXA-181_ (corresponding to *bla*_OXA-1181_), two ATPases, a DUF4158 domain-containing protein, and several intergenic regions. There were two groups within the AM772 plasmids which had zero SNV differences between them; the first contained eight plasmids which were identical to the reference KP400525.1. This group contained plasmids from five different provinces, and were isolated between 2018 and 2021. The second group contained three plasmids isolated from only one province from various species between 2018 and 2020. The remaining plasmids have 1–3 SNVs relative to the others, with no other plasmids containing the same set of SNVs.

Using MOB-recon to search against the clusters generated from our complete and circular OXA-48-type plasmids, we examined the Illumina-only data for remaining *n*=160 (of total 249) isolates. MOB-recon reconstructs plasmids from incomplete Illumina data and we were able to predict plasmid clusters for most isolates (147/160, 91.9 %) when using our complete and circular OXA-48-type plasmids as references. We found 34.4 % (55/160) of isolates with Illumina-only data were predicted to be part of IncX3/rep_cluster_1195 (primary cluster AA836 and secondary cluster AM772) plasmids, providing further support for the prevalence of this plasmid type in Canada. Indeed, between 2019 and 2022, 40 % (55/136) of all OXA-48-type plasmids were from cluster AA836. This type of plasmid was commonly observed in *E. coli* ST410 (18/75, 24.0 %) in our dataset, of which 14 were observed since 2019, indicating this clonal lineage has contributed to this plasmid’s persistence specifically since 2019.

### Canadian plasmids in primary cluster AB871: *bla*_OXA-48_ on IncL/M replicons

The fourteen Canadian plasmids in primary cluster AB871 all grouped in the same secondary cluster AO777. These plasmids varied from 62.8 kb to 69.0 kb in size and were isolated from *E. coli, C. freundii,* and *K. pneumoniae* species complex between 2015 and 2020 from eleven hospital sites in four provinces. No other antimicrobial resistance genes were found on these plasmids aside from *bla*_OXA-48_ within Tn*1999*. These plasmids are >97 % identical to the reference IncL plasmid pOXA-48a (accession JN626286.1) [14,59, 60] with variable insertions or inversions (Fig. S1). These insertion/deletion patterns appear to be random and do not match epidemiological information (year, hospital site, province). The prevalence of secondary cluster AO777 plasmids among multiple genera and provinces along with the presence of conjugation genes (MOBP relaxase and MPF_I mating pair formation protein) suggest that horizontal transmission has contributed to these plasmids’ persistence in Canada.

The Tn*1999* composite transposons are known to encode *bla*_OXA-48_ in IncL/M plasmids [8,19]. We found 71 % (*n*=10/14) of IncL/M plasmids encode the inverted Tn*1999.2*, which is a Tn*1999* variant with IS*1R* inserted into IS*1999* upstream of *bla*_OXA-48_ that has inverted between the *tir* gene insertion site (Table 1) [61,63]. In addition, two plasmids encoded the normal (uninverted) Tn*1999.2*, one encoded an inverted Tn*1999*, and one encoded a modified Tn*1999.2* with a 13 kb insertion.

MOB-recon predicted 20.6 % (*n*=33/160) of isolates generated from Illumina-only data that grouped in this same IncL/M primary (AB871) and secondary cluster (AO777). No plasmid distribution trends across Canada related to hospital site, organism, or type were observed among isolates harbouring these contigs.

### Canadian plasmids in primary cluster AB484: *bla*_OXA-232_ and bla_OXA-181_ on rep_cluster_1195 replicons

The seventeen Canadian plasmids in primary cluster in AB484 all grouped in the same secondary cluster AO082. These plasmids were 6.1 kb with two exceptions at 7.3 kb. All had a replicon identified as rep_cluster_1195 by MOB-suite (also named as ColKP3 by PlasmidFinder [8,59]), and were classified as mobilizable by MOB-suite due to the presence of a MOBP relaxase but did not contain MPF genes. The isolates containing these plasmids contained an average of six plasmids each, which suggests that the 6.1 kb plasmids could perhaps be mobilized by conjugation machinery in co-resident plasmids in those isolates. These plasmids mainly carried *bla*_OXA-232_ (15/17, 88.2 %) with two exceptions carrying *bla*_OXA-181_, and no other resistance genes were found. These plasmids are >99.99 % identical to reference plasmid pOXA-232 first isolated in 2012 (accession JX423831.1) [64]. These plasmids were isolated from *E. coli, R. ornithinolytica,* and *K. pneumoniae* species complex between 2015 and 2020 from thirteen hospital sites in four provinces.

The small 6.1 kb rep_cluster_1195/ColKP3/ColE2 plasmids are associated with Tn*2013* which contains a large deletion on the 5′ end of IS*Ecp1* [8,64]. We found that this structure was preserved in all of our primary cluster AB484 plasmids (Table 1). Many (10/17, 58.8 %) have a single SNV relative to the reference sequence at positions 4282 (1/10), 4283 (1/10), or 4286 (8/10), which corresponds to an intergenic region between Δ*ereA* and *repA*. Additional data on plasmid SNVs did not indicate patterns in space or time.

MOB-recon predicted an additional 5.0 % (*n*=8/160) of isolates generated from Illumina-only data that grouped in this same primary (AB484) and secondary cluster (AO777). This secondary cluster was observed for all *K. pneumoniae* ST231 isolates in our dataset (11/11, 100 %). Indeed, of all *bla*_OXA-232_ observed across Canada, 52.4 % (11/21) were associated with ST231, with no overall epidemiological links.

### Canadian plasmids in primary cluster AC843: *bla*_OXA-204_ on IncC/IncF plasmids

We obtained four complete plasmids which were all classified into primary cluster AC843 and secondary cluster AQ458; all plasmids encoded *bla*_OXA-204_, had IncC/IncFIB/IncFIC/IncFII replicons, were classified as conjugative by MOB-suite, and were found in *C. freundii* ST22 isolated from a single hospital site. We previously characterized an outbreak of *C. freundii* ST22 encoding *bla*_OXA-204_ in Canada from 2016 to 2018 that was attributed to chromosomal genes and three distinct plasmid types [65]. These isolates were not included in our dataset; however, plasmids from this study are part of the global dataset, six of which grouped into the same primary and secondary cluster as the four new *bla*_OXA-204_ plasmids sequenced here (AC843/AQ458). Given the primary cluster assignment is identical between the old and new plasmids, this strain/plasmid combination appears to still be circulating in the same region. On these plasmids, the *bla*_OXA-204_ gene was found within the Tn*6750*, which is a variation of the Tn*2016* transposon with an intact IS*Ecp*1 [8,65]. In addition to encoding *bla*_OXA-204_ on plasmids, these four isolates had a second copy of *bla*_OXA-204_ encoded within a prophage on their chromosomes, which was also observed in the 2016 to 2018 outbreak isolates [65].

A total of 18 isolates in our dataset encoded *bla*_OXA-204_ on complete or incomplete contigs, all of which were *C. freundii* ST22 isolated from the same hospital site in 2016 (*n*=2), 2019 (*n*=7), 2020 (*n*=6) and 2021 (*n*=3). Fourteen of these had Illumina-only data and all but one were predicted to encode *bla*_OXA-204_ on a contig assigned to this same primary cluster (AC843; 17/18, 94.4 %), with the one exception remaining was unclassified. Further investigation is required to confirm these plasmid structures.

Interestingly, no other isolate in our dataset had or was predicted to have a plasmid in this same AQ458 secondary cluster, indicating that this plasmid type has not been observed outside of this region.

### Other *bla*_OXA-48-type_ genes on chromosomes in Canadian isolates

We observed four *bla*_OXA-48-type_ variants on the chromosome in 27 isolates: *bla*_OXA-48_ (*n*=9), *bla*_OXA-244_ (*n*=12), *bla*_OXA-204_ (*n*=4), and *bla*_OXA-181_ (*n*=2). Unlike the other *bla*_OXA-48-type_ variants, *bla*_OXA-244_ was found exclusively on chromosomes and exclusively in *E. coli* in our dataset. Interestingly, *E. coli* ST38 isolates commonly harboured either *bla*_OXA-244_ (*n*=6/12) or *bla*_OXA-48_ (*n*=7/9) on the chromosome.

Chromosomal *bla*_OXA-48_ was associated with Tn*6237*, which is a 21.9 kb IS*1R*-based composite transposon that contains an inverted Tn*1999.2* and a plasmid-derived fragment of pOXA-48a. Tn*6132* is 99.9 % identical to Tn*51098*, which is also a 21.9 kb IS*1R*-based composite transposon but contains *bla*_OXA-244_ [8]. We found Tn*6237* was present in some form in most isolates that had a chromosomal *bla*_OXA-48_ (*n*=8/9) (Table 1); only two contained the full 21.9 kb length, whereas others (*n*=6/9) had a truncated form that was between 10 kb and 20 kb in length. One isolate contained a 3 kb fragment of *lysR* and *bla*_OXA-48_ that was not associated with any other Tn*6237* genes. There was not a conserved genomic locus among isolates where all Tn*6237* genes were inserted, and none of the isolates here had the Tn*6237* insertion in the same region as reference KT444705.1.

Chromosomal *bla*_OXA-244_ genes in varying lengths of Tn*51098* were located at the same genomic locus in all isolates (Fig. S2), all of which were *E. coli* (*n*=12) and represented seven different STs. This genomic locus is the same as reference KR364794.1 [66]. Several isolates had the 21.9 kb full length Tn*51098* match (2/12) (corresponding to genetic environment A in [67]), but the majority had *lysR* interrupted by IS*R1* (7/12) (corresponding to genetic environment G in [67]), resulting in a 3.3 kb truncated fragment. Others had a 15 kb truncated fragment of Tn*51098* (1/12, corresponding to genetic environment D in [67]), a 7 kb truncated fragment of Tn*51098* (1/12), or a duplication of the *bla*_OXA-244_/IS*R1*-truncated *lysR* immediately downstream of the 3.3 kb fragment (1/12).

There were two instances of chromosomal *bla*_OXA-181_ which were found in Tn*1999.3* structures; one was inserted into a fimbrial adhesion operon and the other was integrated at an rRNA/tRNA site.

Some of the incomplete *bla*_OXA-48-type_-encoding contigs were not predicted to be part of any primary plasmid cluster or chromosome (13/160, 8.1 %), some of which (5/13, 38.5 %) were less than 2.3 kb which is close to the length of IS*10A* which makes classification challenging. Further long-read sequencing would be required to verify if the genomic locus of *bla*_OXA-48-type_ genes in these isolates. This method is also unable to predict chromosomal insertions of *bla*_OXA-48-type_ genes, so contigs encoding genes that were primarily predicted to be chromosomal (i.e. *bla*_OXA-244_) but were assigned plasmid clusters should be interpreted with caution.

### Epidemiology of *bla*_OXA-48-type_ plasmids and contigs across Canada

**Fig. 5.**
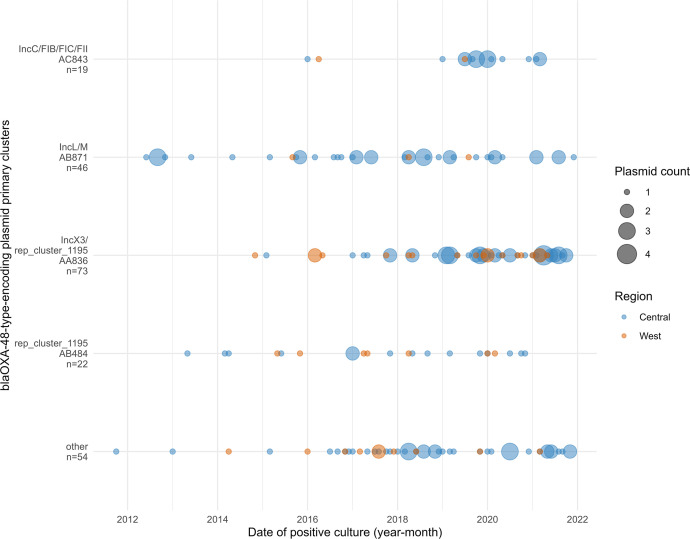
Epidemiology of Canadian OXA-48-type plasmid primary clusters from 2010 to 2021. Date of positive culture was grouped into year-month bins. This data includes both complete circular plasmids and incomplete *bla*_OXA-48-type_-encoding contigs. The Central region represents the provinces of Ontario and Quebec, and the West region represents the provinces of British Columbia, Alberta, and Manitoba. ‘Other’ indicates plasmids which did not group in the top four primary clusters. N indicates total number of isolates in each cluster. Chromosomal *bla*_OXA-48-type_ genes were excluded.

We examined the temporal and geographic patterns of all complete OXA-48-type plasmids and included the predicted OXA-48-type plasmid clusters based off Illumina-only data in Canada from 2010 to 2021 (Fig. 5). Isolates from the central region dominated our dataset (Central: 201/249, 80.7 %; West: 48/249, 19.3 %). Occurrences of *bla*_OXA-48-type_ genes in Canada were low until 2016. Primary cluster AA836 (IncX3/rep_cluster_1195) was the most common plasmid type detected in both West and Central Canada since 2018. Primary cluster AB871 (IncL/M) was one of the first primary clusters that became dominant in Canada and has been isolated sporadically in the Central region since then. Primary cluster AB484 (rep_cluster_1195) plasmids were observed at low frequencies since 2013. Primary cluster AC843 (IncC/FIB/FIC/FII) were detected in 2016 and from 2019 onwards, indicating that this plasmid continues to circulate at this hospital site.

## Discussion

We examined the prevalence and distribution of *bla*_OXA-48-type_-producing *Enterobacterales* and their plasmids in Canada from 2010 to 2021. The majority of OXA-48-type carbapenemase occurrences in Canada during this time period were predominantly from *E. coli* and *K. pneumoniae* species, which agrees with other countries [15,68,70]. This contrasts with *bla*_KPC_, which was found in more diverse genera in Canada during the same time period [71], and with *bla*_NDM_, which also was found in diverse genera around the world [72].

We searched for patterns among plasmid clusters that would allow us to predict dissemination of OXA-48-type plasmids in Canada. We examined SNV prevalence in two primary plasmid clusters whose plasmids were near identical (AA836, AB484). For IncX3/rep_cluster_1195 plasmids carrying *bla*_OXA-181_ (AA836), we found remarkably high conservation among plasmid sequences wherein many were 100 % identical to the reference sequence, agreeing with previous studies [8,15, 69, 73, 74]. Although we found 1–3 SNVs in some plasmid sequences, these were scattered across the genome and did not appear to be linked to any epidemiological information which limits the usefulness of creating a Tn*2013* transposon typing scheme, as done for *bla*_KPC_ genes with Tn*4401* [71,75]. Similarly for the 6.1 kb rep_cluster_1195 plasmids (AB484) plasmids, we observed 0–2 SNVs between plasmids isolated from organisms that have no epidemiological links. Overall, our dataset shows plasmid SNV data is not enough alone to track plasmid spread, and that epidemiological data is critical for tracing transmission of these plasmid clusters due to high levels of sequence conservation.

The IncX3/rep_cluster_1195 (AA836) was the most common plasmid cluster found in Canada. Accordingly, *bla*_OXA-181_ and the Tn*2013* transposon were also the most common in our dataset. This is in contrast to multiple European countries, wherein *bla*_OXA-48_ is the most common *bla*_OXA-48-type_ variant observed paired with IncL/M plasmids [15,68,70, 76].

The IncL/M (AB871) were the second most common primary plasmid cluster in Canada when including plasmids predicted in Illumina-only data (Table 2). David *et al*. [69] observed 82 % of *K. pneumoniae* species complex isolates were predicted to carry IncL/M plasmids in the EUSCAPE study. This incidence is much higher than what we predicted here, wherein only 32.5 % (27/83) of *K. pneumoniae* species complex isolates are predicted to carry an IncL/M pOXA-48a-type plasmid. David *et al.* [69] also showed these plasmids were highly related, with most (76 %) within two SNVs of each other. We observed small-scale recombination and inversions in our plasmids (Fig. S1), which may be explained by larger time gap (2015 to 2020) in our dataset compared to the 2 years (2013–2014) in the David *et al.* publication [69].

There were some clear associations between certain plasmid and *bla*_OXA-48-type_ genes with certain species/sequence types (Fig. 1), where these clones are not restricted to a geographic region/hospital site and have been transmitted across Canada. For example, all *K. pneumoniae* ST231 isolates in this study, which were not linked epidemiologically, encoded *bla*_OXA-232_ on the 6.1 kb rep_cluster_1195 (AB484) plasmids, which has been observed in other countries [18,64, 77]. Similarly, many isolates (18/23, 78.2 %) in the clonal lineage *E. coli* ST410 encoded *bla*_OXA-181_ on IncX3/rep_cluster_1195 plasmids. This multi-drug resistant and diverse lineage has been circulating worldwide with this plasmid for since 2003 [78]. Finally, *E.coli* ST38 was noted in occurrences of *bla*_OXA-244_ (47.4 %) which were all chromosomally integrated and in occurrences of *bla*_OXA-48_ (31.5 %), both on plasmids and chromosomally integrated. *E.coli* ST38 is well documented as a high risk clone and has contributed to the dissemination of *bla*_OXA-48-type_ genes, often by chromosomal integration [79].

Chromosomal integration was observed in 10.8 % of isolates and may be linked to persistence of OXA-48-type carbapenemases in Canada. The persistence of *bla*_OXA-244_ due to chromosomal integration has been suggested by Emeraud *et al.* [67], whom observed a tendency for more recent isolates to encode a truncated form of Tn*51098*, whereas older strains tended to encode a full length transposon. This was also what we observed at the same genomic locus. This truncation suggests that transposition may no longer be possible as *bla*_OXA-244_ becomes a permanent part of the genome. In contrast, the *bla*_OXA-48_ chromosomal insertion sites were not conserved, nor was the length of insertion, which suggests that multiple transposition and recombination events have occurred [15,63].

The ongoing occurrences of *bla*_OXA-204_ in *C. freundii* ST22 at a single hospital site in Canada has not been observed elsewhere in the world [65]. *bla*_OXA-204_ was first detected in *C. freundii* isolated from wastewater samples in Tunisia between 2013 and 2015 [80]. *C. freundii* ST22 isolates encoding *bla*_OXA-204_ were observed in France between 2019 and 2020 but no analysis of the genomic location of the *bla*_OXA-204_ genes was completed [81]. While these isolates comprise only a small proportion of the total *bla*_OXA-48-type_-encoding isolates in this dataset, the longevity of this endemic lineage merits further investigation to determine why this strain/plasmid combination has remained localized and has not spread to other regions in Canada or elsewhere.

Multiple OXA-48-type primary clusters (AA836, AB871, AB484) that are dominant in Canada and spread by horizontal transmission notably lacked other antimicrobial resistance genes. Only *bla*_OXA-48_ was found on IncL/M (AB871) and rep_cluster_1195 (AB484) plasmids, whereas only *bla*_OXA-181_ and *qnrS1* were found on IncX3/rep_cluster_1195 (AA836) plasmids. These findings agree with Hendrickx *et al.* [15], who also observed a lack of antimicrobial resistance genes on *bla*_OXA-48-type_ plasmids in the Netherlands, and with a recent analysis of IncL/M plasmids in NCBI RefSeq which found 82.7 % (162/196) of IncL/M plasmids encoded *bla*_OXA-48_ as the only resistance gene [82]. The lack of antimicrobial resistance genes on OXA-48-type plasmids contrasts with other types of carbapenemase plasmids that frequently have multiple other types of antimicrobial resistance genes, such as *bla*_KPC_ [71] and *bla*_NDM_ genes [72,83].

Overall, our results suggest horizontal transmission (AB871), clonal transmission (AC843), and a combination of both horizontal and clonal transmission (AB484 and AA836) have contributed to OXA-48-type carbapenemase persistence in Canada. Specifically, the emergence of AA836 over the last 4 years indicates a significant driver of OXA-48-type dissemination in Canada. Given that primary cluster AA836 is mobilizable, associated with a wide variety of species, and also associated with the high-risk *E.coli* ST410 lineage, multiple factors are all likely contributing to its persistence. All dominant primary plasmid clusters have been observed elsewhere in the world, with the exception of *bla*_OXA-204_ on IncC/IncF plasmids (AC843) which is unique to Canada. The use of SNV data (AA836, AB484) or trends in plasmid homology (AB871) were not successful in distinguishing top Canadian plasmids by location or time. We suggest maintaining high-quality reference plasmid databases for characterizing OXA-48-type plasmids in new nosocomial isolates. Tracking dissemination of specific *bla*_OXA-48-type_ genes requires plasmid clustering alongside molecular analysis of high-risk lineages in combination with epidemiological data when investigating plasmids with such high sequence homology.

## supplementary material

10.1099/mgen.0.001257 Fig. S1.

10.1099/mgen.0.001257 Fig. S2.

10.1099/mgen.0.001257 Table S1.

## Abbreviations

ARG: antimicrobial resistance gene
MPF: mating pair formation
ONT: Oxford Nanopore Technologies
OXA-48: oxacillinase-48
PTU: Plasmid Taxonomic Unit

## References

[1] Sheu C-C, Chang Y-T, Lin S-Y, Chen Y-H, Hsueh P-R (2019). Infections caused by Carbapenem-resistant *Enterobacteriaceae*: an update on therapeutic options. Front Microbiol.

[2] Queenan AM, Bush K (2007). Carbapenemases: the versatile beta-lactamases. Clin Microbiol Rev.

[3] Karlowsky JA, Lob SH, Kazmierczak KM, Badal RE, Young K (2017). *In vitro* activity of imipenem against Carbapenemase-positive Enterobacteriaceae isolates collected by the SMART global surveillance program from 2008 to 2014. J Clin Microbiol.

[4] Kopotsa K, Osei Sekyere J, Mbelle NM (2019). Plasmid evolution in carbapenemase-producing Enterobacteriaceae: a review. Ann N Y Acad Sci.

[5] Boyd SE, Holmes A, Peck R, Livermore DM, Hope W (2022). OXA-48-like β-Lactamases: global epidemiology, treatment options, and development pipeline. Antimicrob Agents Chemother.

[6] Logan LK, Weinstein RA (2017). The epidemiology of Carbapenem-resistant Enterobacteriaceae: the impact and evolution of a global menace. J Infect Dis.

[7] Canadian Nosocomial infection surveillance program (2022). Healthcare-associated infections and antimicrobial resistance in Canadian acute care hospitals, 2016–2020. Can Commun Dis Rep.

[8] Pitout JDD, Peirano G, Kock MM, Strydom K-A, Matsumura Y (2019). The global ascendency of OXA-48-type carbapenemases. Clin Microbiol Rev.

[9] Poirel L, Potron A, Nordmann P (2012). OXA-48-like carbapenemases: the phantom menace. J Antimicrob Chemother.

[10] Mataseje LF, Boyd DA, Hoang L, Imperial M, Lefebvre B (2013). Carbapenem-hydrolyzing oxacillinase-48 and oxacillinase-181 in Canada, 2011. Emerg Infect Dis.

[11] Lázaro-Perona F, Dahdouh E, Sotillo A, Pérez-Blanco V, Villa J (2022). Dissemination of a single ST11 clone of OXA-48-producing *Klebsiella pneumoniae* within a large polyclonal hospital outbreak determined by genomic sequencing. Microb Genom.

[12] Turton JF, Doumith M, Hopkins KL, Perry C, Meunier D (2016). Clonal expansion of *Escherichia coli* ST38 carrying a chromosomally integrated OXA-48 carbapenemase gene. J Med Microbiol.

[13] Mataseje LF, Abdesselam K, Vachon J, Mitchel R, Bryce E (2016). Results from the Canadian nosocomial infection surveillance program on carbapenemase-producing Enterobacteriaceae, 2010 to 2014. Antimicrob Agents Chemother.

[14] Partridge SR, Kwong SM, Firth N, Jensen SO (2018). Mobile genetic elements associated with antimicrobial resistance. Clin Microbiol Rev.

[15] Hendrickx APA, Landman F, de Haan A, Witteveen S, van Santen-Verheuvel MG (2021). bla OXA-48-like genome architecture among carbapenemase-producing *Escherichia coli* and *Klebsiella pneumoniae* in the Netherlands. Microb Genom.

[16] Honda NH, Aoki K, Kamisasanuki T, Matsuda N, To M (2019). Isolation of three distinct carbapenemase-producing Gram-negative bacteria from a Vietnamese medical tourist. J Infect Chemother.

[17] Carattoli A, Seiffert SN, Schwendener S, Perreten V, Endimiani A (2015). Differentiation of IncL and IncM plasmids associated with the spread of clinically relevant antimicrobial resistance. PLoS One.

[18] Shankar C, Mathur P, Venkatesan M, Pragasam AK, Anandan S (2019). Rapidly disseminating bla_OXA-232_ carrying *Klebsiella pneumoniae* belonging to ST231 in India: multiple and varied mobile genetic elements. BMC Microbiol.

[19] Aubert D, Naas T, Héritier C, Poirel L, Nordmann P (2006). Functional characterization of IS1999, an IS4 family element involved in mobilization and expression of beta-lactam resistance genes. J Bacteriol.

[20] Liu Y, Feng Y, Wu W, Xie Y, Wang X (2015). First report of OXA-181-producing *Escherichia coli* in China and characterization of the isolate using whole-genome sequencing. Antimicrob Agents Chemother.

[21] Clinical and Laboratory Standards Institute (2023). Performance Standards for Antimicrobial Susceptibility Testing: Informational Supplement M100 ED33:2023.

[22] Canadian Nosocomial Infection Surveillance Program (2023). Surveillance Protocol for Carbapenemase-Producing Organisms (CPO) in CNISP Hospitals.

[23] van Domselaar G (2023). RefSeq Masher. https://github.com/phac-nml/refseq_masher.

[24] Lam MMC, Wick RR, Watts SC, Cerdeira LT, Wyres KL (2021). A genomic surveillance framework and genotyping tool for *Klebsiella pneumoniae* and its related species complex. Nat Commun.

[25] Annavajhala MK, Gomez-Simmonds A, Uhlemann A-C (2019). Multidrug-resistant *Enterobacter cloacae* complex emerging as a global, diversifying threat. Front Microbiol.

[26] Brenner DJ, Grimont PA, Steigerwalt AG, Fanning GR, Ageron E (1993). Classification of citrobacteria by DNA hybridization: designation of *Citrobacter farmeri* sp. nov., *Citrobacter youngae* sp. nov., *Citrobacter braakii* sp. nov., *Citrobacter werkmanii* sp. nov., *Citrobacter sedlakii* sp. nov., and three unnamed *Citrobacter* genomospecies. Int J Syst Bacteriol.

[27] Jain C, Rodriguez-R LM, Phillippy AM, Konstantinidis KT, Aluru S (2018). High throughput ANI analysis of 90K prokaryotic genomes reveals clear species boundaries. Nat Commun.

[28] Sutton GG, Brinkac LM, Clarke TH, Fouts DE (2018). *Enterobacter roggenkampii* sp. nov., and *Enterobacter muelleri* is a later heterotypic synonym of *Enterobacter asburiae* based on computational analysis of sequenced Enterobacter genomes. F1000Res.

[29] Mölder F, Jablonski KP, Letcher B, Hall MB, Tomkins-Tinch CH (2021). Sustainable data analysis with Snakemake. F1000Res.

[30] Wick R (2022). Filtlong. https://github.com/rrwick/Porechop.

[31] Wick R (2022). Filtlong. https://github.com/rrwick/Filtlong.

[32] Krueger F, James F, Ewels P, Afyounian E, Schuster-Boeckler B (2021). Trimgalore: V0.6.7. Zenodo.

[33] Andrews S (2020). FastQC. https://www.bioinformatics.babraham.ac.uk/projects/fastqc/.

[34] De Coster W, D’Hert S, Schultz DT, Cruts M, Van Broeckhoven C (2018). NanoPack: visualizing and processing long-read sequencing data. Bioinformatics.

[35] Wick RR, Judd LM, Gorrie CL, Holt KE (2017). Unicycler: resolving bacterial genome assemblies from short and long sequencing reads. PLOS Comput Biol.

[36] Kolmogorov M, Yuan J, Lin Y, Pevzner PA (2019). Assembly of long, error-prone reads using repeat graphs. Nat Biotechnol.

[37] Vaser R, Šikić M (2021). Time- and memory-efficient genome assembly with Raven. Nat Comput Sci.

[38] Li H (2016). Minimap and miniasm: fast mapping and de novo assembly for noisy long sequences. Bioinformatics.

[39] Wick RR, Judd LM, Cerdeira LT, Hawkey J, Méric G (2021). Trycycler: consensus long-read assemblies for bacterial genomes. Genome Biol.

[40] Wick RR, Holt KE (2022). Polypolish: short-read polishing of long-read bacterial genome assemblies. PLoS Comput Biol.

[41] Zimin AV, Salzberg SL (2020). The genome polishing tool POLCA makes fast and accurate corrections in genome assemblies. PLoS Comput Biol.

[42] Bharat A, Petkau A, Avery BP, Chen JC, Folster JP (2022). Correlation between phenotypic and in silico detection of antimicrobial resistance in *Salmonella enterica* in Canada using Staramr. Microorganisms.

[43] Bortolaia V, Kaas RS, Ruppe E, Roberts MC, Schwarz S (2020). ResFinder 4.0 for predictions of phenotypes from genotypes. J Antimicrob Chemother.

[44] Jolley KA, Bray JE, Maiden MCJ (2018). Open-access bacterial population genomics: BIGSdb software, the PubMLST.org website and their applications. Wellcome Open Res.

[45] Seemann T (2023). mlst. https://github.com/tseemann/mlst.

[46] Tonkin-Hill G, MacAlasdair N, Ruis C, Weimann A, Horesh G (2020). Producing polished prokaryotic pangenomes with the Panaroo pipeline. Genome Biol.

[47] Redondo-Salvo S, Bartomeus-Peñalver R, Vielva L, Tagg KA, Webb HE (2021). COPLA, a taxonomic classifier of plasmids. BMC Bioinform.

[48] Robertson J, Nash JHE (2018). MOB-suite: software tools for clustering, reconstruction and typing of plasmids from draft assemblies. Microb Genom.

[49] Robertson J, Bessonov K, Schonfeld J, Nash JHE (2020). Universal whole-sequence-based plasmid typing and its utility to prediction of host range and epidemiological surveillance. Microb Genom.

[50] Kurtz S, Phillippy A, Delcher AL, Smoot M, Shumway M (2004). Versatile and open software for comparing large genomes. Genome Biol.

[51] Siguier P, Perochon J, Lestrade L, Mahillon J, Chandler M (2006). ISfinder: the reference centre for bacterial insertion sequences. Nucleic Acids Res.

[52] Petkau A, Mabon P, Sieffert C, Knox NC, Cabral J (2017). SNVPhyl: a single nucleotide variant phylogenomics pipeline for microbial genomic epidemiology. Microb Genom.

[53] Arndt D, Grant JR, Marcu A, Sajed T, Pon A (2016). PHASTER: a better, faster version of the PHAST phage search tool. Nucleic Acids Res.

[54] R Core Team (2022). R Foundation for Statistical Computing.

[55] Wickham H, Averick M, Bryan J, Chang W, McGowan L (2019). Welcome to the tidyverse. JOSS.

[56] Pedersen TL (2022). patchwork: the composer of plots. https://cran.r-project.org/web/packages/patchwork/index.html.

[57] Kassambara A (2023). Ggpubr: ‘Ggplot2’ based publication ready plots. https://cran.rproject.org/web/packages/ggpubr/index.html.

[58] Schmartz GP, Hartung A, Hirsch P, Kern F, Fehlmann T (2022). PLSDB: advancing a comprehensive database of bacterial plasmids. Nucleic Acids Res.

[59] Carattoli A, Zankari E, García-Fernández A, Voldby Larsen M, Lund O (2014). In silico detection and typing of plasmids using PlasmidFinder and plasmid multilocus sequence typing. Antimicrob Agents Chemother.

[60] Poirel L, Bonnin RA, Nordmann P (2012). Genetic features of the widespread plasmid coding for the carbapenemase OXA-48. Antimicrob Agents Chemother.

[61] Carrër A, Poirel L, Eraksoy H, Cagatay AA, Badur S (2008). Spread of OXA-48-positive carbapenem-resistant *Klebsiella pneumoniae* isolates in Istanbul, Turkey. Antimicrob Agents Chemother.

[62] Sattler J, Tsvetkov T, Stelzer Y, Schäfer S, Sommer J (2022). Emergence of Tn 1999.7, a new transposon in BLA OXA-_48 -har_boring plasmids associated with increased plasmid stability. Antimicrob Agents Chemother.

[63] Beyrouthy R, Robin F, Delmas J, Gibold L, Dalmasso G (2014). IS1R-mediated plasticity of IncL/M plasmids leads to the insertion of bla OXA-48 into the *Escherichia coli* chromosome. Antimicrob Agents Chemother.

[64] Potron A, Rondinaud E, Poirel L, Belmonte O, Boyer S (2013). Genetic and biochemical characterisation of OXA-232, a carbapenem-hydrolysing class D β-lactamase from Enterobacteriaceae. Int J Antimicrob Agents.

[65] Gobeille Paré S, Mataseje LF, Ruest A, Boyd DA, Lefebvre B (2020). Arrival of the rare carbapenemase OXA-204 in Canada causing a multispecies outbreak over 3 years. J Antimicrob Chemother.

[66] Potron A, Poirel L, Dortet L, Nordmann P (2016). Characterisation of OXA-244, a chromosomally-encoded OXA-48-like β-lactamase from *Escherichia coli*. Int J Antimicrob Agents.

[67] Emeraud C, Girlich D, Bonnin RA, Jousset AB, Naas T (2021). Emergence and polyclonal dissemination of OXA-244-producing *Escherichia coli*, France. Emerg Infect Dis.

[68] Findlay J, Perreten V, Poirel L, Nordmann P (2022). Molecular analysis of OXA-48-producing *Escherichia coli* in Switzerland from 2019 to 2020. Eur J Clin Microbiol Infect Dis.

[69] David S, Cohen V, Reuter S, Sheppard AE, Giani T (2020). Integrated chromosomal and plasmid sequence analyses reveal diverse modes of carbapenemase gene spread among *Klebsiella pneumoniae*. Proc Natl Acad Sci U S A.

[70] Patiño-Navarrete R, Rosinski-Chupin I, Cabanel N, Zongo PD, Héry M (2022). Specificities and commonalities of carbapenemase-producing *Escherichia coli* isolated in France from 2012 to 2015. mSystems.

[71] Lerminiaux N, Mitchell R, Bartoszko J, Davis I, Ellis C (2023). Plasmid genomic epidemiology of blaKPC C_Arb_Apenemase-producing Enterobacterales in Canada, 2010–2021. Antimicrob Agents Chemother.

[72] Nordmann P, Poirel L, Walsh TR, Livermore DM (2011). The emerging NDM carbapenemases. Trends Microbiol.

[73] Yu Z, Zhang Z, Shi L, Hua S, Luan T (2022). In silico characterization of IncX3 plasmids carrying blaOXA-181 in Enterobacterales. Front Cell Infect Microbiol.

[74] Mouftah SF, Pál T, Darwish D, Ghazawi A, Villa L (2019). Epidemic IncX3 plasmids spreading carbapenemase genes in the United Arab Emirates and worldwide. Infect Drug Resist.

[75] Sheppard AE, Stoesser N, German-Mesner I, Vegesana K, Sarah Walker A (2018). TETyper: a bioinformatic pipeline for classifying variation and genetic contexts of transposable elements from short-read whole-genome sequencing data. Microb Genom.

[76] Zwittink RD, Wielders CC, Notermans DW, Verkaik NJ, Schoffelen AF (2022). Multidrug-resistant organisms in patients from Ukraine in the Netherlands, March to August 2022. Euro Surveill.

[77] Mancini S, Poirel L, Tritten M-L, Lienhard R, Bassi C (2018). Emergence of an MDR *Klebsiella pneumoniae* ST231 producing OXA-232 and RmtF in Switzerland. J Antimicrob Chemother.

[78] Roer L, Overballe-Petersen S, Hansen F, Schønning K, Wang M (2018). *Escherichia coli* sequence type 410 is causing New International High-Risk Clones. mSphere.

[79] Notermans DW, Schoffelen AF, Landman F, Wielders CCH, Witteveen S (2022). A genetic cluster of OXA-244 carbapenemase-producing *Escherichia coli* ST38 with putative uropathogenicity factors in the Netherlands. J Antimicrob Chemother.

[80] Sghaier S, Abbassi MS, Pascual A, Serrano L, Díaz-De-Alba P (2019). Extended-spectrum β-lactamase-producing Enterobacteriaceae from animal origin and wastewater in Tunisia: first detection of O25b-B2_3_-CTX-M-27-ST131 Escherichia coli and CTX-M-15/OXA-204-producing Citrobacter freundii from wastewater. J Glob Antimicrob Resist.

[81] Biez L, Bonnin RA, Emeraud C, Birer A, Jousset AB (2023). Nationwide molecular epidemiology of carbapenemase-producing *Citrobacter* spp. in France in 2019 and 2020. mSphere.

[82] Fordham S, Mantzouratou A, Sheridan EA (2022). Bioinformatic analyses of plasmid resistome changes in pOXA-48. Microbiology.

[83] Dong H, Li Y, Cheng J, Xia Z, Liu W (2022). Genomic epidemiology insights on NDM-producing pathogens revealed the pivotal role of plasmids on *bla*_NDM_ transmission. Microbiol Spectr.

