# Supplementary material for: Plasmid genomic epidemiology of carbapenem-hydrolysing class D β-lactamase (CDHL)-producing Enterobacterales in Canada, 2010−2021
Source: Microb Genom. 2024 Jun 19;10(6):001257. doi: 10.1099/mgen.0.001257 (PMC11261825; doi:10.1099/mgen.0.001257)
Supplement: Fig. S1. [file mgen-10-01257-s001.pdf]

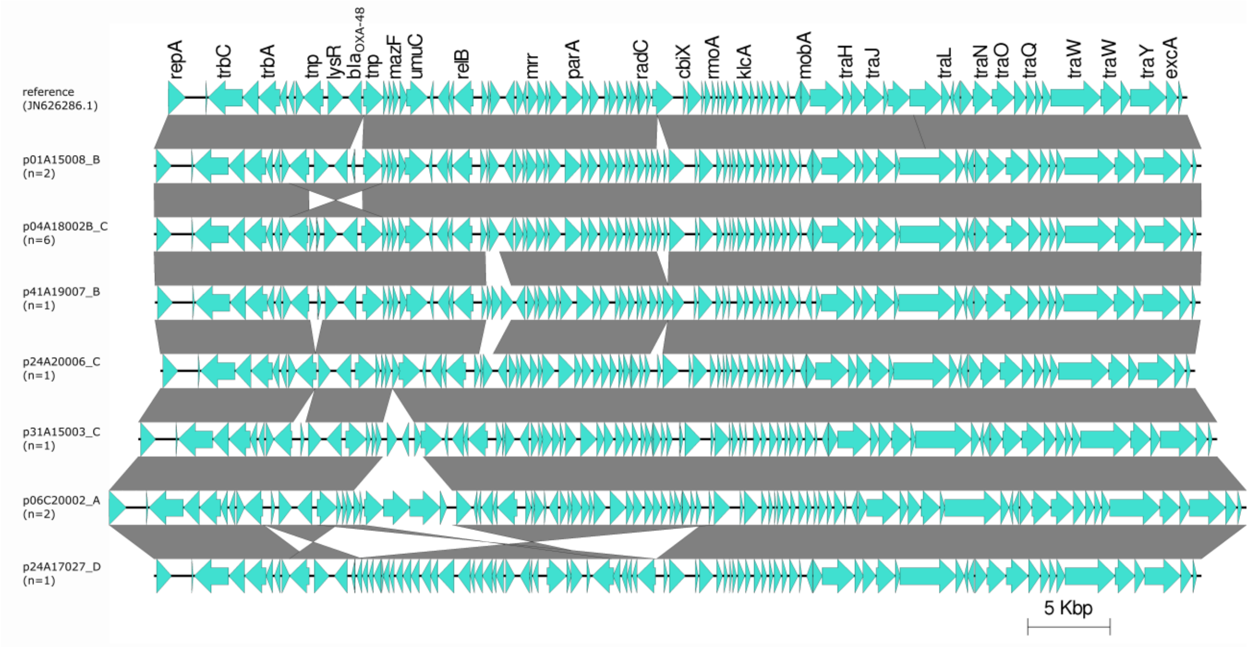

Supplementary Figure 1. Variations in complete *bla*$_{OXA-48}$ IncL/M (AB871/AO777, n=14) Canadian plasmid structure compared to the reference pOXA-48a plasmid (JN626286.1, top track). One plasmid was chosen as representative for each structure where multiple identical structures existed, and "N" indicates the number of plasmids sequenced here that have identical structures. Grey boxes between gene tracks indicate 100% percent identity via blastn search between adjacent tracks.
