# Supplementary material for: Plasmid genomic epidemiology of carbapenem-hydrolysing class D β-lactamase (CDHL)-producing Enterobacterales in Canada, 2010−2021
Source: Microb Genom. 2024 Jun 19;10(6):001257. doi: 10.1099/mgen.0.001257 (PMC11261825; doi:10.1099/mgen.0.001257)
Supplement: Fig. S2. [file mgen-10-01257-s002.pdf]

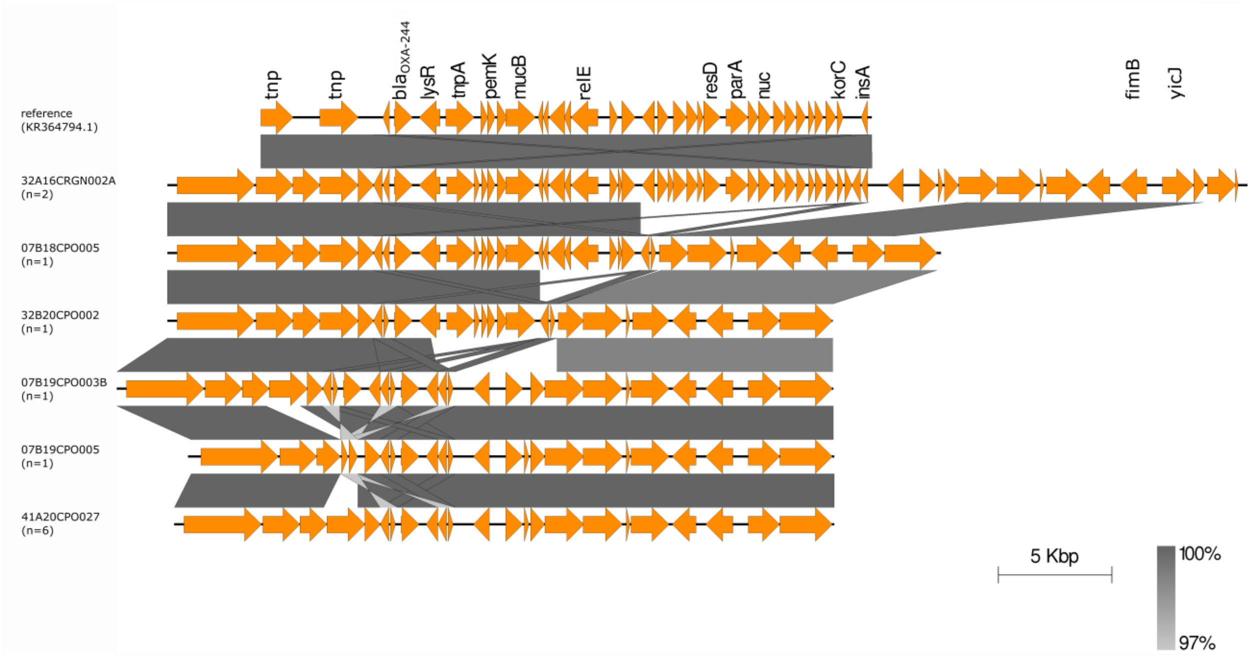

Supplementary Figure 2. Structure of conserved *bla*$_{OXA-244}$ chromosomal insertion sites in *E. coli* (n=12). Structures among Canadian plasmids were compared to the reference (KR364794.1, top track). One chromosome was chosen as representative for each structure where multiple identical structures existed, and "N" indicates the number of plasmids sequenced here that have identical structures. Grey boxes between gene tracks indicates percent identity via blastn search between adjacent tracks.
